# Changes in serum proteins after endotoxin administration in healthy and choline-treated calves

**DOI:** 10.1186/s12917-016-0837-y

**Published:** 2016-09-20

**Authors:** Z. Yilmaz, O. Eralp Inan, M. Kocaturk, A. T. Baykal, O. Hacariz, I. Hatipoglu, A. Tvarijonaviciute, M. Cansev, J. Ceron, I. H. Ulus

**Affiliations:** 1 Department of Internal Medicine, Faculty of Veterinary Medicine, Uludag University, 16059 Bursa, Turkey; 2 Medical and Surgical Experimental Animal Practice and Research Center, Eskisehir Osmangazi University, 26480 Eskisehir, Turkey; 3 Department of Medical Biochemistry, Acibadem University School of Medicine, Istanbul, Turkey; 4 TÜBİTAK Marmara Research Center, Genetic Engineering and Biotechnology Institute, Kocaeli, Turkey; 5 Arthritis & Clinical Immunology Research Program, Oklahoma Medical Research Foundation, Oklahoma, USA; 6 Departament de Medicina i Cirurgia Animals, Universitat Autònoma de Barcelona, 08193 Bellaterra, Barcelona, Spain; 7 Department of Pharmacology, Uludag University School of Medicine, Bursa, Turkey; 8 Interdisciplinary Laboratory of Clinical Analysis, Interlab-UMU, Regional Campus of International Excellence “Campus Mare Nostrum”, University of Murcia, Espinardo, Murcia 30100 Spain; 9 Department of Pharmacology, Acibadem University School of Medicine, Istanbul, Turkey

**Keywords:** Calves, Choline, Endotoxemia, Proteomic, Sepsis

## Abstract

**Background:**

This study aimed to investigate the possible serum protein changes after endotoxin administration in healthy and choline-treated calves using proteomics. These results are expected to contribute to the understanding of the pathophysiological mechanisms of endotoxemia and the beneficial effect of choline administration in this clinical situation.

**Methods:**

Healthy-calves (*n* = 20) were divided into 4 groups: Control, Choline treated (C), Lipopolysaccharide administered (LPS), and LPS + C. Control calves received 0.9 % NaCl injection. Calves in C and LPS + C groups received choline chloride (1 mg/kg/iv). Endotoxin (LPS) was injected (2 μg/kg/iv) to the calves in LPS and LPS + C groups. Serum samples were collected before and after the treatments. Differentially expressed proteins (> 1.5 fold-change relative to controls) were identified by LC-MS/MS.

**Results:**

After LPS administration, 14 proteins increased, and 13 proteins decreased within 48 h as compared to controls. In the LPS group, there were significant increases in serum levels of ragulator complex protein (189-fold) and galectin-3-binding protein (10-fold), but transcription factor MafF and corticosteroid binding globulin were down regulated (≥ 5 fold). As compared with the LPS group, in LPS + C group, fibrinogen gamma-B-chain and antithrombin were up-regulated, while hemopexin and histone H4 were down-regulated. Choline treatment attenuated actin alpha cardiac muscle-1 overexpression after LPS.

**Conclusions:**

LPS administration produces changes in serum proteins associated with lipid metabolism, immune and inflammatory response, protein binding/transport, cell adhesion, venous thrombosis, cardiac contractility and blood coagulation. The administration of choline is associated with changes in proteins which can be related with its beneficial effect in this clinical situation.

**Electronic supplementary material:**

The online version of this article (doi:10.1186/s12917-016-0837-y) contains supplementary material, which is available to authorized users.

## Background

Endotoxemia is defined as the presence of endotoxins in blood. This situation can occur by Gram-negative bacterial infections which liberate endotoxin (lipopolysaccharides; LPS) during rapid growth. This results in the fact that Gram-negative sepsis is associated with high mortality rates, despite comprehensive treatment in intensive care patients [1]. Studies are underway with regards to understanding the complex pathophysiological mechanism of endotoxemia, describing criteria for early diagnosis and developing new treatment approaches in order to decrease mortality [2, 3].

Intravenous choline administration show beneficial effects in the treatment of endotoxemia [4–7] in various species such as dogs and rats. Beneficial effects of intravenous choline treatment are reported in association with the activation of the efferent vagus nerve-based cholinergic anti-inflammatory pathway by the increase in nicotinic cholinergic neuro-transmission [5, 8] following enhanced acetylcholine release by choline [9]. However the possible changes in serum proteins that choline treatment can produce has not been studied.

Proteomics analysis allows the simultaneous analysis of thousands of proteins in a sample. This could facilitate the identification of new biomarkers of use for diagnosis and prognosis of sepsis/endotoxemia [10, 11]. Thus, proteomic analyses are a growing trend in human [12] and veterinary medicine [13]. In particular proteomic studies allow identifying new proteins that change in endotoxemia, and by knowing the function of these new proteins, new mechanisms involved in endotoxemia could be elucidated and therefore increase the knowledge about its pathophysiology [14].

In the light of our previous findings [4–7], we hypothesize that the beneficial effect of choline administration in endotoxemic patients could be mediated by changes in concentrations of selected serum proteins. Therefore the objective of this study was to evaluate the possible changes that can occur in serum proteins, by a proteomic analysis, after endotoxin administration in healthy and choline-treated calves.

## Methods

### Experimental set up

Healthy Holstein calves (4 weeks of age; 40 kg mean weight; *n* = 20) were used in the present study. Their health status was evaluated by clinical, haematological and serum biochemical analyses. Calves were negative for bovine herpes virus, bovine leucosis virus, brucellosis, bovine viral diarrhea, foot and mouth disease, infectious bovine rhinotracheitis, paratuberculosis, and tuberculosis when tested by commercial ELISA kits (IDVet Diagnostics, Grabels, France).

Calves were randomized equally into 4 groups: Control, Choline treated (C), Lipopolysaccharide administered (LPS), and LPS + C. Calves in the Control group were injected with 0.9 % NaCl (saline; 5 ml, i.v.) twice at an interval of 5 min while those in C group received choline chloride (1 mg/kg, dissolved in 5 ml of saline, i.v., once), 5 min after 0.9 % NaCl administration (5 ml, i.v.). In the LPS group, endotoxin (LPS) was injected (2 μg/kg, dissolved in 5 ml of normal saline, i.v., once) to the calves, followed 5 min later by intravenous injection of normal saline. In LPS + C group, endotoxin injection was followed 5 min later by choline chloride injection at the doses described in the LPS and C groups, respectively [15, 16].

### Clinical data

Routine clinical examinations were performed before (baseline) and 0.5–48 h after the treatments. A scoring system adapted from a previous paper [17] was used to evaluate clinical status in the study. Each parameter was scored by minimum 0 point (means normal) to maximum 6 points (means severely affected).

### Serum proteomic analysis

In order to analyse differences in protein expression in the course of the study, three serum samples at each time point for each treatment were obtained and the protein profiles of the serum samples were analysed by LC-MS/MS method [18–20]. Albumin depletion from serum samples was done with albumin depletion spin columns (Pierce, Thermo Scientific). IgGs from the albumin-depleted serum samples were also depleted with an Albumin/IgG Removal Kit (Pierce, Thermo scientific). The remaining serum proteins (at same concentration for all samples) were extracted and enzymatically digested into peptides, and the resulting peptides were subjected to the nanoUPLC-ESI-qTOF-MS system (nano-ultra-performance LC and ESI quadrupole TOF MS) (Waters) with the application of data independent acquisition method also known as the MS^E^ as previously described in detail [20]. Minimum 3 technical replicates for each sample were used for the analysis. The proteomic data was searched against a reviewed database [including protein sequences of *Bos taurus* and an internal standard (enolase, *Saccharomyces cerevisiae*; #P00924), obtained from UniProt (htpp://www.uniprot.org)] with previously described *in silico* parameters [19]. Quantitative differences of proteins at different time points were calculated using Progenesis LC-MS software V4.0 (Nonlinear Dynamics) [20].

### Chemicals

Choline chloride and endotoxin (*E. coli* lipopolysaccharide, LPS, serotype 055:B5) were purchased from Sigma Chemical Co. (St. Louis, Missouri, USA).

### Statistical analysis

Results were expressed as mean ± SEM. Data were evaluated by one-way analysis of variance by repeated measures, followed by Tukey test for pairwise comparisons. Clinical scores were compared by a non-parametric test (Friedman Repeated Measure ANOVA on Ranks). *P* values less than 0.05 were considered significant.

## Results

### Clinical data

**Fig. 1:**
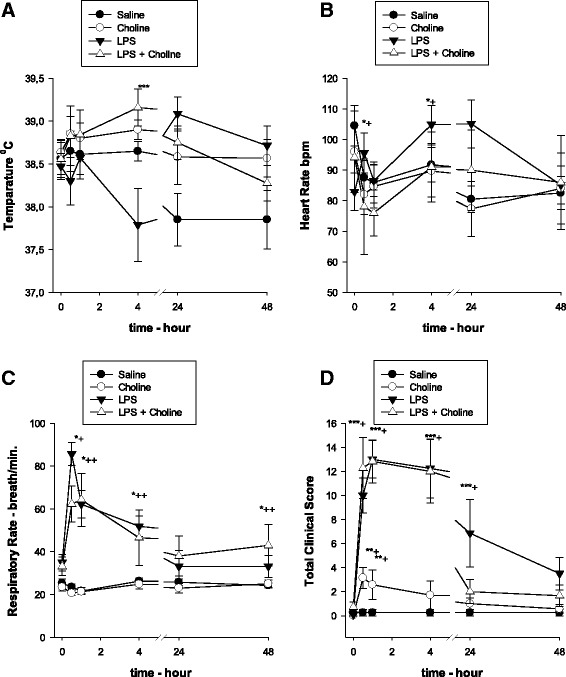
Clinical parameters (**a**, temperature; **b**-**c**, heart and respiratory rates; and **d**, total clinical score) in saline, choline, LPS and LPS + choline groups. Calves were injected i.v. once with saline (5 ml) or saline containing choline chloride (1 mg/kg). Five minutes after the first i.v. treatment, calves received endotoxin (LPS, 2 μg/kg) in 5 ml normal saline. Measurements were performed immediately before first i.v. treatment (0 h) and 1 – 48 h after saline, choline, LPS or LPS + choline treatments. Each point represents to Mean ± SEM of six determinations. * *p* < 0.05, **p < 0.001, ***p < 0.001 as compared to baseline + *p* < 0.05, ++*p* < 0.01 as compared to saline group

Clinical findings are presented as Additional file 1 (Fig. 1). Briefly, choline induced a decrease in heart and respiratory rates compared with the control animals. LPS administration increased body temperature and heart and respiratory rates as compared to their baselines. The severities of these changes in LPS + C treatment were lower (*p* < 0.01) than those of calves treated with LPS.

Two calves died at 4 and 24 h after LPS and one at 48 h after LPS + C. Calves in control and choline groups survived until the end of the study.

### Serum proteins

Protein expression differences among experimental groups at different time points (30 min, 1, 4 and 48 h) are shown in Table 1. Details including numerical values for the quantitative analysis obtained from the Progenesis LC-MS software are shown in Additional file 1.

**Table 1:** Protein expression differences among experimental groups at different time points (30 min, 1 h, 4 h and 48 h)

LPS_Choline	P02081; Hemoglobin fetal subunit beta/1.52 Q9QAQ7; Non structural protein of 4 9 kDa/3.42 P02676; Fibrinogen beta chain/3.83 A2I7N1; Serpin A3 5/1.44 Q3ZC07*; Actin alpha cardiac muscle 1/15.21 Q32L76; Serum amyloid A 4 protein/1.70 A2I7N3; Serpin A3 7/1.44 Q3ZC07*; Actin alpha cardiac muscle 1/187.33 P0CH28*; Polyubiquitin C/1.59 *P12799; Fibrinogen gamma B chain/8.44* *Q2M2T2; U-p-C19orf71-h/2.17* *P02676; Fibrinogen beta chain/64.53*	A2I7N1; Serpin A3 5/1.44 Q9TTK6; Membrane primary amine oxidase/Infinity P01966; Hemoglobin subunit alpha/1.50 P19034; Apolipoprotein C II/2.82 Q9MYP6; beta hydroxysteroid dehydrogenase 14/26.44 Q1JQ97; Bardet Biedl syndrome 4 protein homolog/2.30 *Q9TTK6; Membrane primary amine oxidase/11.79* *P81644; Apolipoprotein A II/1.45* *P19035; Apolipoprotein C III/2.34* *Q32L76; Serum amyloid A 4 protein/2.20* *Q5E9E3; Complement C1q subcomponent subunit A/3.01* *P34955; Alpha 1 antiproteinase/1.60* *P28800; Alpha 2 antiplasmin/1.43* *P80012; von Willebrand factor Fragment/2.98*	Q9TTE1; Serpin A3 1/2.09 Q80E01; Glutaredoxin 1/2.21 Q3SZJ0; Argininosuccinate lyase/1.81 Q17QL1; Glucosamine 6 phosphate isomerase 2/2.96 Q3T0Z5; Leukocyte receptor cluster m-1-h/2.22 Q9TTE1; Serpin A3 1/1.74 Q9TTK6; Membrane primary amine oxidase/64.58 *A2I7N0; Serpin A3 4/1.47* *P41361; Antithrombin III/2.67* *P00735; Prothrombin/2.287* *P12799; Fibrinogen gamma B chain/5.92*	
LPS	P17697; Clusterin/1.43 *Q0V898; Negative elongation factor E/1.68* *Q9TTK6; Membrane primary amine oxidase/7.17* *A6QPQ2; Serpin A3 8/1.67* *O46406; Primary amine oxidase lung isozyme/3.74* *P01044; Kininogen 1/2.13* *A7E3W2; Galectin 3 binding protein/13.09* *P41356; Fusion glycoprotein F0/1.82* *O62830-2; Isoform Beta 2 of Protein phosphatase 1B/6.78* *Q3T132; Ragulator complex protein LAMTOR2/189.56* *Q3SZJ0; Argininosuccinate lyase/9.22* *Q9QAQ7; Bovine coronavirus strain OK 0514 GN 4a/4.93* *Q0V898; Negative elongation factor E/10.86* *Q28901;6-p-2-k-f-2-6-b-f/Infinity*	A2I7N2; Serpin A3 6/1.64 P81644; Apolipoprotein A II/1.62 P19035; Apolipoprotein C III/2.80 Q5E9E3; Complement C1q subcomponent subunit A/2.66 Q28901; 6-p-2-k-f-2-6-b-f/infinity *P01966; Hemoglobin subunit alpha/1.81* *P02081; Hemoglobin fetal subunit beta/1.55* *P80109; P-g-s-p-d/1.50* *Q5E9F5; Transgelin 2/2.90* *P22226; Cathelicidin 1/13.17* *Q7SIH1; Alpha 2 macroglobulin/1.41* *A2I7M9; Serpin A3 2/9.68* *P00924; ENO1 YEAST Enolase 1/1.46* *Q3MHN5; Vitamin D binding protein/1.67* Q3ZC07*; Actin alpha cardiac muscle 1/25.52 *P80109; Phosphatidylinositol glycan specific phospholipase D/3.11* *O02659; Mannose binding protein C/5.76* P0CH28*; Polyubiquitin C/4.09 *A8YXX7; Trefoil factor 3/4.93* *Q2TBS3; Uncharacterized protein C20orf79 homolog/4.76* *Q96629; Adenain/2.23* *P19034; Apolipoprotein C II/4.21* *Q1JP73; UPF0553 protein C9orf64 homolog/12.57*		Q9TTK6-2; Isoform 2 of Membrane primary amine oxidase/2.30 Q1JP73; UPF0553 protein C9orf64 homolog/1.95 Q56K14; 60S acidic ribosomal protein P1/Infinity *Q3SZV7; Hemopexin/2.10* *Q1JQ97; Bardet Biedl syndrome 4 protein homolog/1.48* *Q56K14;60S acidic ribosomal protein P1/Infinity* *Q96629; Adenain/1.74* *Q2KIT0; Protein HP 20 homolog/1.93* *Q5E9F5; Transgelin 2/Infinity* *P62803; Histone H4/11.67*
Choline	O46406; Primary amine oxidase lung isozyme/1.74 Q28901; 6-p-2-k-f-2-6-b-f/Infinity *Q2M2T2; Uncharacterized protein C19orf71/1.78* *A2I7N3; Serpin A3 7/1.56* *Q17QL1; Glucosamine 6 phosphate isomerase 2/1.48*		*A5PJC4; Ubiquitin ISG15 c. e. E2 L6/1.45* *Q80E01; Glutaredoxin 1/1.40* *Q0VCI2; Syntaxin 19/3.81*	Q0V898; Negative elongation factor E/2.4 Q05204; Lysosome associated membrane glycoprotein 1/4.03 Q56K14; 60S acidic ribosomal protein P1/8968.4 *P34955; Alpha 1 antiproteinase/1.47* *A2I7N2; Serpin A3 6/1.72* *Q3SZJ0; Argininosuccinate lyase/1.41* *Q17QL1; Glucosamine 6 phosphate isomerase 2/8.44*
Control		P80012; von Willebrand factor Fragment/2.08	A2I7N0; Serpin A3 4/1.60	P21752; Thymosin beta 10/1.45
		Q3SZK0; Solute carrier family 25 member 34/1.72	Q2TBS3; Uncharacterized protein C20orf79/2.01	Q3T132; Ragulator complex protein LAMTOR2/2.04
		Q05204; Lysosome associated membrane glycoprotein 1/2.34		P01044-2*; Isoform LMW of Kininogen 1/2.87
			*Q9TTK6-2; Isoform 2 of Membrane primary amine oxidase/2.82*	
		*Q80E01; Glutaredoxin 1/3.37*		Q58CQ9; Pantetheinase/1.58
		*Q05204; Lysosome associated membrane glycoprotein 1/10.89*	*Q3MHN5; Vitamin D binding protein/1.48*	
				Q96629; Adenain/1.69
		*Q2KIU3; Protein HP 25 homolog 2/1.70*	*P56652; Inter alpha trypsin inhibitor heavy chain H3/1.58*	
		*P02081; Hemoglobin fetal subunit beta/1.72*	*Q29443; Serotransferrin/1.57*	P01044-2*; Isoform LMW of Kininogen 1/1.62
			*Q32PJ2; Apolipoprotein A IV/1.45*	*Q58CQ9; Pantetheinase/1.84*
			*A2I7N2; Serpin A3 6/4.57*	*P21752; Thymosin beta 10/1.46*
			*Q2KJF1; Alpha 1B glycoprotein/1.46*	*Q9MYP6;17 beta hydroxysteroid dehydrogenase 14/2.24*
			*P01966; Hemoglobin subunit alpha/1.63*	
			*P02070; Hemoglobin subunit beta/1.98*	
			*A7YY73; Transcription factor MafF/5.00*	
			*E1BF81; Corticosteroid binding globulin/5.27*	
	Control	Choline	LPS	LPS_Choline

This table shows the identified proteins with highest mean condition (left) and lowest mean condition (bottom) among experimental groups at different time points at statistically significant level (*P* < 0.05). Relative abundance levels of the proteins with highest mean condition, compared to the proteins with lowest mean condition, are indicated after the slash (/). Time points are indicated with underline, italic and italic + underline, for 1 h, 4 h and 48 h time points, respectively. Asterisk (*) indicates the proteins, of which, isoforms are detectable (see Additional file for the accession numbers of the isoforms). Abbreviations; c. e.: conjugating enzyme, 6-p-2-k-f-2-6-b-f; 6 phosphofructo 2 kinase fructose 2 6 bisphosphatase 3 Fragment, m-1-h; member 1 homolog, U-p-C19orf71-h; Uncharacterized protein C19orf71 homolog, P-g-s-p-d; Phosphatidylinositol glycan specific phospholipase D

A total of 76 proteins were identified across the serum samples by the proteomic analysis. After LPS administration, 14 proteins increased, whereas 13 proteins decreased within 48 h as compared to controls (Table 1). In LPS group, there was a dramatic increase (189 fold, at 24 h) in ragulator complex protein LAMTOR2 which was followed by other proteins showing changes ranging from 6 to 13 fold such as negative elongation factor E (at 4 h), galectin-3 binding protein (Gal-3BP), argininosuccinate lyase and membrane primary amine-oxidase (at 48 h). Following LPS administration, moderate decreases (< 5 fold changes) were observed in some proteins such as serpin A3-4 (at 30 min.), vitamin D binding protein (VDBP), inter alpha trypsin inhibitor heavy chain H3 (ITIH, at 4 h), apolipoprotein A IV (Apo-AIV), alpha-1B glycoprotein and serotransferrin (at 48 h). In addition more prominent decreases (≥ 5 fold changes, at 48 h) were observed in transcription factor MafF and corticosteroid binding globulin (CBG).

When compared with LPS group, in LPS + C group, glucosamine-6-phosphate isomerase-2, membrane primary amine oxidase, fibrinogen gamma B chain, antithrombin and prothrombin were markedly up-regulated, while isoform 2 of membrane primary amine oxidase, hemopexin and histone-H4 were markedly down-regulated. Actin alpha cardiac muscle-1 expression increased at 1–48 h (15–187 fold) in LPS and LPS + C groups, as compared to controls. The magnitude of increase in actin alpha cardiac muscle-1 was lower at 48 h in LPS + C group than that of LPS group. As compared to controls, following choline administration, a number of proteins (primary amine oxidase, serpin A3-7 and glucosamine-6-phosphate isomerase-2) were up-regulated while others (lysosome associated membrane glycoprotein-1 and glutaredoxin-1) were more down-regulated at different time points of the study.

## Discussion

In this study, an experimental model consisting in the induction of endotoxemia by LPS administration in calves was chosen, because calves are very sensitive to LPS. Therefore is expected that the changes associated with LPS as well as its treatment will be easier to detect than with other animal models. The clinical changes occurred after LPS treatment in our experimental model in calves showed similarities with the results of experimental human [21] and animal studies of sepsis/endotoxemia [4–7, 15–17].

To our knowledge, our study describes for the first time 14 proteins that are up-regulated, and 13 proteins that are down-regulated in endotoxemia in calves. The roles of some of these altered proteins are highlighted below.

One of the proteins that were dramatically up-regulated was ragulator complex protein LAMTOR2. This protein is known to be involved in amino acid sensing and activation of mTORC1 [22], a signalling complex promoting cell growth in response to growth factors, energy levels, and amino acids. A previous study reported that severe immunological defects affecting the immunity in human primary immunodeficiency syndrome was associated with reduced levels of LAMTOR2 [22]. Hence, the elevated serum levels of ragulator complex in our study may be associated with host’s response by increasing macrophage activity and enhancing adaptive immunity in response to LPS. Thus, ragulator complex may represent a new early diagnostic marker for the detection of inflammation in calves, due to its dramatic overexpression shortly after LPS treatment.

Another protein, which significantly increased in calves after LPS administration, was Gal-3BP. This protein has stimulatory activity on lymphokine-activated and natural killer cells, and also stimulates the secretion of many cytokines and interleukins in peripheral blood mononuclear cells that all play a contributory role in inflammation [23]. In this study, up-regulated expression of Gal-3BP may be associated with limiting an on-going inflammation in response to LPS, because of its immune modulatory activity [23]. In addition, overexpressed Gal-3BP may be considered as a host response to neutralize harmful effects of Galectin-3 which is released from LPS-stimulated cells to produce heightened levels of inflammatory mediators, resulting in further tissue damage and, ultimately, organ failure, characteristics of sepsis. On the other hand, Gal-3BP has a critical role in the development of venous thrombosis [24] and to increase the survival of cancer cells in the bloodstream [25], indicating that probably is involved in the pathophysiological responses of different pathological conditions.

Transcription factor MafF and CBG are associated with interleukin regulation [26], and have a protective effect in situations of cell stress, coagulation cascade activation and severe illness [27]. A possible reason for the down regulations of these proteins found in our study may be excessive use of them to neutralize detrimental effects of LPS during acute phase reaction.

Proteases from LPS-activated leukocytes can trigger tissue and organ damage and enhance the nonspecific proteolysis of plasma clotting factors in patients with severe sepsis. One example is the leukocyte elastase that is involved in the progress of complications in patients with sepsis [28]. In the present study, down regulation of ITIH-H3 in calves with LPS was most probably due to extended secretion of elastase [28], thereby this protein may be a part of the regulatory system that controls acute inflammation [29] in calves. Similar to our finding, circulating ITIH protein level was found lower in patients with severe sepsis than in healthy volunteers [28].

Confirming our previous findings in dogs and rats [4–7], we found in the present study that choline treatment improved the clinical signs associated to LPS. Further studies including larger number of calves are needed to evaluate if choline treatment could reduce mortality rate in this clinical condition. Endotoxemia studies in dogs showed that these beneficial effects of choline treatment might be related with inhibition of TNF-α synthesis [4], prevention of hepato-renal injury [4], and attenuation of the changes of serum butrylcholinesterase and paraoxonase-1 activities [30].

Most of the down-regulated proteins after choline treatment such as kininogen-1, prothrombin, and ragulator complex are involved in coagulation and the development of DIC. Therefore the decrease of these proteins could be associated with the preventive effects of choline treatment on the development of coagulopathy, as described previously in dogs [7]. In addition the down-regulation of histones after choline treatment would be related with the protective effect of choline. Histone proteins, mainly the histones-H3 and H4, have been reported to exhibit cytotoxicity when released to the extracellular fluid in response to severe stress or inflammatory challenges like sepsis and to mediate excessive and overwhelming cell damage and death [31]. Hemopexin (Hx) is mainly expressed as an APP from liver after inflammation [32]. In this study, Hx overexpression may be related with its antioxidant role [32] to facilitate tissue repair in response to LPS [33]. Choline treatment decreased Hx overexpression in calves with LPS, probably related with the reduction in body’s antioxidant needs [32, 34] due to the reduction in tissue damage produced by choline [3].

The increase found in SAA4 after choline administration could be related with the protective effect that has been described for SAA by inhibiting TNF and LPS as well as platelet aggregation [35]. In addition a correlation between SAA4 and cholinesterase has been found, therefore a link between SAA4 response and choline administration could be hypothesized [36].

Choline treatment attenuated overexpression of contractile protein actin alpha cardiac muscle-1 (ACTC1) at 48 h after LPS, indicating a protective effect of choline treatment on sepsis-induced myocardial dysfunction (SIMD). SIMD is a well-recognized manifestation of organ dysfunction in patient with sepsis [37] and calves with endotoxemia [38]. LPS-induced overexpression of ACTC1 may contribute to myocardial dysfunction during endotoxemia [39], most probably due to impaired sarcomere integrity [40]. The observed overexpression of ACTC1 may be due to decreased thymosin beta-10 expression since thymosin beta-10 plays an important role in the organization of the cytoskeleton by binding to and sequestering actin monomers resulting with an inhibition in actin polymerization [41].

## Conclusion

 Although this is a pilot study made with a small sample size and therefore the results should be taken with caution. It could be concluded that based on our experimental model, following LPS administration, there are changes in selected serum proteins which are associated with various biological processes such as lipid metabolism, immune and inflammatory response, protein binding and transport, cell adhesion, venous thrombosis, cardiac contraction and blood coagulation. In addition choline administration is associated with changes in serum concentrations of selected serum proteins that would be related with its beneficial effect in this clinical situation. These findings would reinforce the effectiveness of choline administration in the treatment of endotoxemia. However further studies are needed to confirm our results due to small sample size of this study, and in addition it would be interesting to evaluate the effectiveness of choline treatment in combination with other drugs such as steroidal or non-steroidal anti-inflammatory drugs.

## Additional file

Additional file 1: Clinical parameters (temperature, heart and respiratory rates, and total clinical score) in all groups. (XLSX 109 kb)
